# Integration of molecular and computational approaches paints a holistic portrait of obscure metabolisms

**DOI:** 10.1128/mbio.00431-23

**Published:** 2023-10-19

**Authors:** Victor Reyes-Umana, Sophia D. Ewens, David A. O. Meier, John D. Coates

**Affiliations:** 1 Department of Plant and Microbial Biology, University of California, Berkeley, California, USA; Albert Einstein College of Medicine, Bronx, New York, USA

**Keywords:** (per)chlorate, perchlorate, phosphite, iodate, chlorate, dissimilatory

## Abstract

Microorganisms are essential drivers of earth’s geochemical cycles. However, the significance of elemental redox cycling mediated by microorganisms is often underestimated beyond the most well-studied nutrient cycles. Phosphite, (per)chlorate, and iodate are each considered esoteric substrates metabolized by microorganisms. However, recent investigations have indicated that these metabolisms are widespread and ubiquitous, affirming a need to continue studying the underlying microbiology to understand their biogeochemical effects and their interface with each other and our biosphere. This review focuses on combining canonical techniques of culturing microorganisms with modern omic approaches to further our understanding of obscure metabolic pathways and elucidate their importance in global biogeochemical cycles. Using these approaches, marker genes of interest have already been identified for phosphite, (per)chlorate, and iodate using traditional microbial physiology and genetics. Subsequently, their presence was queried to reveal the distribution of metabolic pathways in the environment using publicly available databases. In conjunction with each other, computational and experimental techniques provide a more comprehensive understanding of the location of these microorganisms, their underlying biochemistry and genetics, and how they tie into our planet’s geochemical cycles.

## INTRODUCTION

At the intersection of microbiology and geochemistry is the interdisciplinary subjects of biogeochemistry and geomicrobiology, whose origins can be traced back to the mid-1800s (1). Sergei Winogradsky exemplified this early work through his research and descriptions on the role of sulfur oxidation in elemental sulfur precipitation (2), the role of iron oxidation in both ferric oxide deposits and bicarbonate geochemistry (3), and the influence of nitrifying bacteria on nitrogen availability in agricultural practices (4). In concert with other scientists of this era, Winogradsky laid the intellectual groundwork for microbiologists to identify and characterize novel metabolisms and their interactions with global geochemical cycles (5). Microorganisms drive these nutrient cycles through either assimilation (biomass accumulation) or dissimilation (energy production). Dissimilatory pathways are essential to biogeochemical cycles because metabolized substrates and end products are immediately exchanged with the environment rather than integrated into biomass (6, 7).

Extensive work on primary nutrient cycles (like carbon, nitrogen, and sulfur) shows the diversity of mechanisms that microorganisms use to transform these elements for energy generation. Ultimately, microbial biotransformation influences the surrounding geochemistry in geologically and anthropogenically relevant ways. Carbon is perhaps the most widespread and influential cycle in Earth’s biogeochemical system (8). Most carbon fixation pathways are exclusively used for carbon assimilation, but the Wood-Ljungdahl pathway is also used for energy conservation via CO_2_ dissimilation (9). Like carbon, nitrogen is highly redox active, and bioavailable compounds include nitrate (NO_3_^−^) and nitrite (NO_2_^−^) oxyanions, as well as dinitrogen (N_2_) and reduced ammonium (NH_4_^+^) species (10). Multiple biotic processes have been observed interfacing with these nitrogen species, including nitrification, denitrification, ammonification, ammonia oxidation, and nitrogen fixation, which collectively influence agricultural nitrogen availability, eutrophication, and marine community composition (10). Meanwhile, sulfur undergoes biotransformations that include (among many alternative variations) sulfate (SO_4_^2−^) reduction, sulfide (HS^−^) oxidation, and sulfur (S^0^) disproportionation (11, 12), all of which serve as mechanisms for biological energy conservation and have simultaneous geochemical and engineering consequences like hydrogen-sulfide corrosion, metal-sulfide precipitation, sulfide toxicity, and sulfur precipitation (13). The complex global cycles of carbon, nitrogen, and sulfur are well studied, and because of their broad range of oxidation states, these elemental cycles are influenced by microbial redox reactions (Table 1).

**TABLE 1:** Redox potentials of various electron donors, electron acceptors, and common cofactors involved in microbial electron transfers at pH 7^*e*^

Element/category	Equation (ox/red)	Reduction potential (mV)	Reference
Phosphorus (P)	H_2_PO_2_^−^/P_4_	1,220	(14)
	HPO_3_^2−^/PH_3_	760	(14)
	HPO_3_^2−^/H_2_PO_2_^−^	740	(14)
	H_2_PO_2_^−/^PH_3_	660	(14)
**→**	H_2_PO_4_^−^/HPO_3_^2−^	650	(14)
	P_4_/PH_3_	480	(14)
			
Hydrogen (H)	H^+^/H_2_	414	(15)
			
Sulfur (S)	SO_4_^2−^/HSO_3_^−^	516	(15)
	S_2_O_3_^2−^/HS^−^ + HSO_3_^−^	402	(15)
	S^0^/HS^−^	270	(15)
	HSO_3_^−^/S_3_O_6_^2−^	173	(15)
	HSO_3_^−^/HS^−^	116	(15)
	S_3_O_6_^2−^/S_2_O_3_^2−^ + HSO_3_^−^	+225	(15)
			
Carbon (C)	CO_2_/formate	432	(15)
	CO_2_/acetate	290	(15)
	CO_2_/CH_4_	244	(15)
	Acetaldehyde/ethanol	197	(15)
	Pyruvate^−^/lactate^−^	190	(15)
	Dihydroxyacetone phosphate/glycerol-phosphate	190	(15)
	Xaloacetate^2−^/malate^2−^	173	(15)
	Glycine/acetate^−^ + NH_4_^+^	10	(15)
	Fumarate/succinate	+33	(15)
			
Transition metals	Mo (E'_01_)^*c*^^,*d*^	265	(16)
	Mo^5+^/Mo^4+^	18	(16)
	Mo^6+^/Mo^5+^	+110	(16)
	Fe^3+^/Fe^2+^	+772	(15)
			
Nitrogen (N)	NO_2_^−^/NO	+350	(15)
	NO_3_^−^/NO_2_^−^	+433	(15)
	NO/N_2_O	+1,175	(15)
			
Iodine (I) →	IO_3_^−^/I^−^	**+**720^*a*^	(17)
			
Oxygen (O)	O_2_/H_2_O	+818	(15)
			
Chlorine (Cl) →	ClO_3_^−^/ClO_2_^−^	+744	(18)
**→**	ClO_4_^−^/ClO_3_^−^	+813	(18)
**→**	ClO^−^/Cl^−^	+1,261	(18)
**→**	ClO_2_^−^/ClO^−^	+1,280	(18)
			
Cofactors^*b*^	F_430_:Ni^2+^/Ni^+^	650	(19)
	Cob(II)alamin/cob(I)alamin; *base-off*	572	(20)
	Cob(II)alamin/cob(I)alamin	526	(20)
	Ferredoxin ox/red (E'_01_)	398	(15)
	F_420_ ox/red	380	(21)
	Flavodoxin ox/red (E'_01_)	371	(15)
	NAD/NADH	320	(15)
	Cytochrome c3 ox/red	290	(15)
	FAD/FADH_2_	220	(15)
	FMN/FMNH_2_	190	(15)
	Flavodoxin ox/red (E'_02_)	115	(15)
	Menaquinone ox/red	74	(15)
	APS/AMP + HSO_3_^−^	60	(15)
	Rubredoxin ox/red	57	(15)
	Acrylyl-CoA/propionyl-CoA	15	(15)
	2-Demethylvitamin K_2_ ox/red	+25	(15)
	Ubiquinone ox/red	+113	(15)
	Cob(III)alamin/cob(II)alamin	+273	(20)
	Cu^2+^/C^0^	+341	(22)

^*a*^ Reduction potential reported at pH 8.

^*b*^ The reduction potential of cofactors is highly variable, depending on their coordination state in the associated protein.

^*c*^ E'_01_ signifies a single electron transfer.

^*d*^ The referenced study did not assign the precise oxidation states of the Mo atom.

^*e*^ For a more complete collation of reduction potentials, the reader is referred to Bard et al. (23).

The diversity of microbial energy metabolisms extends beyond the most well-studied global nutrient cycles, often involving comparatively rare substrates in the environment. Studies continually unveil esoteric dissimilatory microbial metabolisms, that herein, we denote dark energy metabolisms (DEMs), which utilize elements spanning the periodic table, including non-metals (24, 25), metalloids (26), transition metals (27), actinides (27), and rare earth elements (1, 28) and play an important role in their geochemical cycle. Substrates for DEMs support microbiology on Earth and reciprocally influence the host and environment. As increasingly diverse microbial metabolisms are identified, it is crucial to characterize their influence on the respective elemental cycles. Here, we briefly examine the current state of understudied DEM using phosphite, perchlorate, chlorate [collectively referred to herein as (per)chlorate], and iodate oxyanions as examples. We summarize how interdisciplinary approaches were used to study these metabolisms and propose a harmonized methodology for understanding new DEMs. Our proposed method seeks to aid in developing more precise biogeochemical models that incorporate esoteric metabolites and will mutually enable the study of the diverse newly recognized microorganisms central to these metabolic processes.

## PHOSPHOROUS, CHLORINE, AND IODINE DEMS

### Phosphite and the global P cycle

Phosphorous (P) is essential for all life on Earth and is a necessary element in biological systems. Four significant phenomena define biogeochemical P models: (i) tectonic uplift, which supplies terrestrial systems with buried P; (ii) erosion and weathering of rocks, which expose dissolved and particulate P to local water systems; (iii) transport of P to the ocean via rivers; and (iv) oceanic sedimentation of P associated with organic and mineral matter that is then returned to terrestrial systems via tectonic uplift (29). In these models, “P” encompasses mineral P, inorganic P, and organic P, all of which are assumed to contain oxidized P (P^5+^) (30). However, environmental phosphorus exists in alternative, reduced valence states that are largely excluded from geochemical models (31). For example, phosphite (P^3+^) has been detected in diverse environments at concentrations ranging from 0.1 to 1.3 µM, accounting for 1% to 33% of total dissolved P in these systems (21, 22, 24). Estimates also suggest that the magnitude of reduced P produced in the ocean exceeds the magnitude of oxidized P delivered via riverine transport, supporting the notion that current P models do not appropriately account for the influence of reduced P species like phosphite (29).

The exclusion of reduced P from geochemical models is likely due to our limited understanding of its neogenesis. While reduced P was likely prevalent on Archean Earth, the Great Oxygenation Event (~2.5 billion years ago; Gya) would have rendered it negligible in contemporary environments due to its abiotic oxidation to phosphate on geological timescales (32). Yet reduced P continues to be detected in diverse neoteric environments (21, 22, 24). Most of these environments are anoxic and reducing, but up to 1 mM phosphite has been observed in surface waters, which suggests contemporary neogenesis because ancient reserves of reduced P would be oxidized in surface waters on geological timescales (14). Though the primary modes of reduced P neogenesis have yet to be confirmed, several mechanisms have been hypothesized. Some reduced P is likely due to anthropogenic activity, as phosphite is a byproduct of industrial phosphonate production (33), and phosphite itself is used as a reducing agent in electroplating (34) and as a fungicide in agriculture (35). Indeed, phosphite is detected in wastewater (36, 37) and higher concentrations of phosphite are observed in heavily polluted lakes compared to less impacted areas (38, 39). In pristine systems, geothermal and hydrothermal activity may generate phosphite through metal phosphide corrosion and iron-mediated phosphate reduction (40, 41). Phosphite might also be produced by the partial oxidation of phosphine (O_2_ and/or UVB), which is a ubiquitous gas at low concentrations and has been measured at higher concentrations in highly reduced sediments, organically enriched wastes (e.g., animal manure), and animal flatus (42). The frequency with which phosphine is detected in sites of high microbial activity contributed to the controversial claim that phosphine in the cloud decks of Venus indicated the presence of life (43, 44). While this claim is highly speculative and largely contested, it has sparked intense debate that has highlighted our limited knowledge of P redox transformations and their relationship to life processes (45–48). Furthermore, the undefined mechanisms for phosphine biogenesis provide little opportunity to integrate phosphite neogenesis into the larger P redox cycle. Alternatively, studies have suggested that phosphite may be derived from biological phosphonate degradation or anomalous phosphate reduction (49–51). However, none of the hypotheses for biological phosphite generation have been experimentally validated, and the enigma of contemporary, environmental (bio)synthesis of most reduced P species is yet to be resolved.

Dissimilatory phosphite oxidation (DPO) is likely the principal mechanism by which localized phosphite is converted to phosphate at biologically relevant timescales since abiotic phosphite oxidation is a slow process—even under hot (95°C) aerobic conditions, phosphite remains stable for months (32). The only known energy metabolism to utilize electrons from phosphorus is DPO, whereby phosphite (P^3+^) serves as the electron donor and energy source for chemolithotrophic bacterial growth (31). Phosphite is theoretically the most energetically favorable chemotrophic electron donor known due to the extremely negative reduction potential of the phosphate/phosphite couple (E^o′^ = −650 mV) (14). With its high solubility and chemical stability, the low redox potential enables phosphite to drive cellular growth through DPO, resulting in phosphate excretion as a product of energy metabolism (25).

The capacity to perform DPO is attributed to the *ptx-ptd* gene cluster (52, 53), with a maximum of seven genes (*ptxDE-ptdFCGHI*) whose synteny depends on the host’s phylogenetic lineage (54). Most *ptx-ptd* genes have not been functionally characterized; however, thermodynamic and physiological evidence suggests that every phosphite oxidized generates one NADH and one ATP (25, 54, 55). All but one of the known dissimilatory phosphite-oxidizing microorganism (DPOM) genomes lack a canonical, membrane-bound electron transport chain, suggesting that DPOM generate ATP via substrate-level phosphorylation and regenerate NADH via an uncharacterized mechanism (54, 55). The only exception to this is the DPOM isolate *Desulfotignum phosphitoxidans,* which shows clear evidence of the involvement of horizontal gene transfer for this metabolism. Physiology and metagenomics studies suggest that CO_2_ is the primary electron acceptor for DPO energy conservation, and genomic analyses suggest that autotrophic DPOM principally use the reductive glycine pathway for CO_2_ fixation and reduction (54, 55). In 2000, *Desulfotignum phosphitoxidans* FiPS-3 was the first DPOM to be isolated, and *Phosphitivorax anaerolimi* Phox-21 was identified approximately 15 years later in an enrichment metagenome (25, 55). The scarcity of DPOM representatives in conjunction with the enigmatic prevalence of phosphite reserves originally implied that DPO metabolism was rare. However, emerging evidence challenges this assumption, as selective enrichments, genome-resolved metagenomic sequencing, and metagenomic data mining strategies have expanded known DPOM diversity and prevalence (54). DPOM are now known to exist globally in anoxic environments, spanning six taxonomic classes that include the monoderm (Gram positive) and diderm (Gram negative) bacteria (54). This proposed taxonomic diversity was recently validated with the isolation of a second DPOM, *Phosphitispora fastidiosa*, confirming the existence of the Gram-positive DPOM (56) previously predicted through genome-resolved metagenomic analysis (54). Phylogenetic analyses have also shown that *ptx-ptd* genes form a monophyletic clade whose evolutionary history is dominated by vertical inheritance likely originating from an ancient microbial ancestor ~3.2 Gya (54). This proposed ancient origin is consistent with the hypothesized prevalence of reduced P on Archean Earth (32), and DPOM could have proliferated until the GOE, after which point, they have likely been maintained in those pockets where phosphite continues to be available (54).

### (Per)chlorate and the global chlorine cycle

Chlorine (Cl) is one of the most abundant rock-forming elements on Earth, with a predicted abundance of 60 × 10^21^ and 26 × 10^21^ g of elemental chlorine in the crust and oceans globally (57). It exists in various oxidation states (Cl^1−^ to Cl^7+^) ranging from its most oxidized form (ClO_4_^−^) to the most reduced forms (chloride and organochlorides). The most abundant form of chlorine is the reduced chloride (Cl^−^) ion, which is essential for various biological processes across life (18, 58) Chlorine cycling through the atmosphere and hydrosphere is potentiated by biological activity in marine environments or by anthropogenic activity (59). Many biological processes interface with chloride ions to form a medley of organochlorine compounds in terrestrial and marine environments (60). Chlorination of organic molecules is a widespread phenomenon that occurs either non-specifically by free hypohalous acids produced by chloroperoxidases or specifically via FADH_2_-dependent halogenases that are broadly present in biosynthetic gene clusters (61). Chlorides and organochlorides from marine and terrestrial environments subsequently enter the atmosphere where they undergo either dry deposition (gases or particles with Cl^−^) or wet deposition (precipitation with dissolved Cl^−^) (62, 63). It is believed that atmospheric wet deposition of perchlorate is widespread and accounts for the high concentrations of perchlorate in arid regions such as the Atacama Desert, Antarctic dry valleys, and parts of the Southwestern United States (63–65). In the atmosphere, Cl compounds react photochemically with a variety of oxidants (e.g., O_3_) or via electrical discharge to form perchlorate and chlorate species [collectively described hereafter as (per)chlorate] (66, 67). Despite being a strong oxidant, perchlorate is stable and non-volatile at ambient temperatures (68, 69). Consequently, anthropogenic contamination of groundwater with (per)chlorate often results in persistently high concentrations of perchlorate specifically, ranging from 630,000 to 3,700,000 µg L^−1^ at sites where ammonium perchlorate was manufactured as a rocket propellant (70). Contamination of groundwater impacts human health through the direct consumption of contaminated groundwater or indirectly through vegetables irrigated with contaminated water. Interest in understanding the fate of (per)chlorate has coincided with identifying organisms capable of reducing these chlorine oxyanions to innocuous chloride. These organisms form an integral part of the chlorine biogeochemical cycle and can potentially play a vital role in the bioremediation of (per)chlorate from contaminated ecosystems.

Dissimilatory microbial (per)chlorate reduction (DPR) was first identified in 1928 when researchers found that microorganisms could degrade the (per)chlorate salts in herbicide (71). Following this observation, researchers believed that reduction was an artifact of nitrate reductase activity (72). However, Romanenko et al. isolated the bacterium *Vibrio dechloraticans* Cuznesove B-1168, which coupled anaerobic growth to reducing perchlorate and chlorate into chloride (73). Early descriptions of subsequent (per)chlorate-reducing microorganisms (DPRM) focused primarily on their unique energy conservation mechanism. Following this, *Acinetobacter thermotoleranticus* (74) was isolated; however, the earliest isolate described in detail was *Ideonella dechloratans* (75), although subsequent studies demonstrated that it was only capable of chlorate reduction and not perchlorate reduction. In the following years, strain GR-1 and *Wolinella succinogenes* HAP-1 were isolated from a similar environment (76, 77). Notably, Rikken et al. demonstrated that strain GR-1 would oxidize acetate as an electron donor proportional to the (per)chlorate provided as electron acceptors and that the chlorite (ClO_2_^−^) concentration increased proportional to the consumption of perchlorate, but chlorite transformation occurred independently of acetate oxidation (76). This led to the proposal that intracellular chlorite is detoxified via disproportionation to molecular oxygen (O_2_) and chloride (Cl^−^), which was later demonstrated to be a highly conserved step across all canonical (per)chlorate respiring species (78). Protein purification subsequently confirmed the existence of the chlorite dismutase (Cld), which serves as the crucial final step in canonical dissimilatory (per)chlorate reduction (78, 79). Chlorite detoxification is the growth limiting step of (per)chlorate reduction, requiring the presence of a mechanism for chlorite detoxification (80, 81). Canonical DPRM detoxify chlorite using Cld and reduce the oxygen liberated from chlorite dismutation via a cbb3 cytochrome oxidase (82). Cryptic perchlorate reducers detoxify chlorite through a Cld-independent process involving the abiotic oxidation of chemical-reducing agents such as HS^−^ or ferrous iron (Fe(II)) with the resultant production of chloride and elemental sulfur (S^o^) or ferric iron (Fe(III)), respectively (82, 83). Alternatively, in symbiotic perchlorate reduction, organisms lacking the *cld* gene can respire perchlorate through exchange of chlorate or chlorite biogenic intermediates, which are subsequently consumed by a second organism (84, 85). As in canonical perchlorate reduction, the partner organism that dismutates chlorite with Cld also respires the oxygen generated (85). While the environmental significance of cryptic perchlorate reduction warrants further investigation as this metabolism has only been observed in the laboratory, studies have suggested that, in some environments, symbiotic perchlorate reduction may be the dominant microbial strategy involved in perchlorate removal (84).

All initial DPRM were enriched from municipal wastewater treatment facilities (73–77), and (per)chlorate reduction was thought to occur as a consequence of anthropogenic contamination. However, the ubiquity of DPRM was established with the isolation and characterization of 20 new microorganisms from both contaminated and pristine sediments that were capable of oxidizing a broad range of organic and inorganic electron donors (78, 86). The recognized phylogenetic diversity of DPRM has since expanded significantly across the Proteobacteria phylum including the Alpha-, Beta-, Gamma-, Delta-, and Epsilonproteobacteria. The most active families are primarily found in the Rhodocyclaceae, Pseudomonadaceae, Campylobacteraceae, and Sedimenticolaceae (86–88), and the best studied isolates are Betaproteobacteria (86). The phylogenetic breadth of DPR exists because the genetic underpinnings of the (per)chlorate reductase pathways are horizontally transferred (89, 90). However, the mechanisms by which either the perchlorate-reducing- or chlorate-reducing gene cluster mobilized are significantly different. Chlorate reduction in various Betaproteobacteria is likely mobilized on a plasmid, as the genes are components of composite transposons (91). The chlorate reduction composite transposon interior (CRI) consists of the core *clrABCD* genes and a chlorite dismutase (*cld*), separately flanked by repetitive DNA, and is mobilized separately (91). Dissimilatory chlorate reduction also relies on host electron transport chains and cofactor biosynthesis genes, as the genes encoding these functions are missing from the composite transposons. The perchlorate reduction island (PRI) is mobilized using different mechanisms from CRI, likely involving Xer recombination and integrative and conjugative elements (87). However, PRI hosts are taxonomically similar to hosts of the CRI, and the horizontally transferred genes include the *pcrABCD* and the *cld*. Notably, while PRIs are distributed among several different Proteobacteria, they are rarely transferred between classes of bacteria and instead undergo frequent transfer in phylogenetically constrained groups (87).

Both chlorate reductase and perchlorate reductase belong to the type II DMSO reductase superfamily of proteins that includes the eponymous dimethylsulfoxide reductase and the nitrate reductase (80). Both enzymes share a common ancestor with all other type II DMSO reductases; however, the perchlorate reductase (PcrA) is phylogenetically closer to the dissimilatory nitrate reductase NarG than it is to the chlorate reductase (ClrA) (80). The extended CRI gene cluster of chlorate reducers (e.g., *Shewanella algae* ACDC) generally consists of nine genes, including the α, β, and γ subunits of the periplasmically expressed chlorate reductase (Clr) and other auxiliary genes like *clrD* (82, 90). By comparison, the PRI gene cluster consists of four genes encoding the α and β subunits of the periplasmic perchlorate reductase (Pcr), a c-type cytochrome, and a chaperone protein. Auxiliary proteins associated with the PRI genes typically include a methionine-rich peptide MrpX and YedYZ, which likely defend the cell against reactive chlorine species (81, 92). The mechanism of perchlorate reduction involves electrons from the quinone pool being transferred to a specific quinone oxidoreductase (PcrQO) and subsequent transfer to a soluble periplasmic c-type cytochrome (PcrC) that interacts with the soluble periplasmic β-subunit of the perchlorate reductase (PcrAB) reducing perchlorate to chlorite (82, 87). All known (per)chlorate-reducing bacteria are facultative aerobes or microaerophilic, with some able to reduce nitrate, and while perchlorate-reducing bacteria can alternatively reduce chlorate, by contrast chlorate-reducing bacteria using an evolutionarily distinct reductase cannot reduce perchlorate (70, 82).

### Iodate and the global iodine cycle

Iodine (I) is unique among elemental cycles as it exists almost entirely in marine and marine-proximal environments, predominating as either iodate (IO_3_^−^) or iodide (I^−^). It is highly soluble and variably sorbed onto soils or sediments. In the absence of biological interactions, environmental hydrology primarily influences its mobility and fate, resulting in iodine accumulation in oceans (93, 94). In seawater, dissolved iodine concentrations average 0.45 μM (95). However, thermodynamic calculations predict that the IO^3−^:I^−^ concentration ratio should be 3.2 × 10^13^ in oxygenated seawater (pH 8.05, pε 12.5) (93). Although iodine should exist predominantly as IO_3_^−^ in fully oxygenated seawater (93), iodide dominates surface seawater at significant concentrations (50–150 nM), reaching 0.3 µM in zones with high biological productivity (96–99). Geochemical analyses reveal that global oceanic iodine concentrations are relatively consistent. Still, the iodine speciation (IO_3_^−^ or I^−^) is variable and correlates to environmental parameters. Due to its antioxidant properties, iodine (as I^−^) is often bioconcentrated in algae and marine life by several orders of magnitude above seawater levels. Kelps, such as *Laminaria digitata*, are known to bioconcentrate iodine and can accumulate up to 50 mM iodide in their tissues (100). Similarly, marine sediments rich in organic matter serve as an iodine sink, reaching concentrations upward of 200 µg per gram of sediment (101). This is severalfold the concentration found in the open ocean and produces volatile iodine species (VOIs) such as methyl iodide (102). VOIs contribute to the destruction of tropospheric ozone (a significant greenhouse gas) and nucleate aerosols in the marine boundary layer, increasing albedo (103–105). Despite the global biological and geochemical significance of iodine, little is known about the microbial impact linking the biogeochemistry of this cycle (106).

Scientists have known for more than 50 years that bacteria can reduce IO_3_^−^ (87). The high reduction potential (IO_3_^−^/I^−^*E_h_* = 0.72 V at pH 8.1) (93, 107) makes IO_3_^−^ an ideal electron acceptor for microbial metabolism. Early studies by Tsunogai and Sase indicated that numerous microorganisms, including *Escherichia coli* and *Shewanella putrefaciens,* reduce IO_3_^−^ to I^−^ (87, 102). Researchers hypothesized that a promiscuous nitrate reductase mediated this metabolism, and subsequent biochemical studies demonstrated that endogenous nitrate and perchlorate reductases could reduce IO_3_^−^ *in vitro* (80, 92).

Multiple mechanisms have been attributed to the reduction of iodate to iodide. For instance, indirect biological iodate reduction was demonstrated in cell suspensions of *Desulfovibrio desulfuricans* and *Shewanella putrefaciens*, which reduced up to 100 µM iodate under anaerobic conditions (108). Both organisms reduce ferric iron and sulfur species to form ferrous iron and sulfide, respectively, causing concomitant reduction of IO_3_^−^ (109). Additional studies affirm that byproducts of anaerobic respiration such as bisulfide (110) and ferrous iron (111) also reduce iodate. *Shewanella putrefaciens* MR-4 reduces IO_3_^−^ independently of sulfide production, and its activity was attributed to iodate consumption in an Arabian Sea oxygen minimum zone (112). Pure cultures of MR-4 showed the removal of 250 µM IO_3_^−^ with equimolar production of I^−^ when lactate was present, suggesting that *Shewanella* uses iodate as a terminal electron acceptor (112). Ten additional IO_3_^−^-reducing *Shewanella* strains were identified recently and shown to reduce IO_3_^−^ independent of the nitrate reductase (NapA) (113). A follow-up study found proteins associated with the extracellular electron conduit (EEC), specifically MtrA and MtrB, conferring the IO_3_^−^ reduction phenotype, suggesting a possible role for the EEC in iodate reduction broadly (114). Cell senescence of numerous phytoplankton has also been implicated in iodate reduction, with some suggesting that intracellular sulfur-containing molecules reduce iodate upon release from a dying cell (115, 116). This observation laid the groundwork for the follow-up study by Bluhm et al*.,* demonstrating that cell senescence drove iodate reduction in multiple algal species (117). Lastly, both promiscuous and dedicated type-II DMSO enzymes have recently been shown to reduce IO_3_^−^ to I^−^ (17, 80, 118). In bacteria, like *Agrobacterium*/*Rhizobium* strain DVZ35, iodate and nitrate are concurrently reduced when nitrate is present, suggesting that a nitrate reductase is responsible for IO_3_^−^ reduction (119). The perchlorate reductase (PcrAB) in *Azospira oryzae* GR-1 and *Azospira suillum* PS shows *in vitro* activity against IO_3_^−^, suggesting DMSO reductases may broadly turn over the molecule as well (80, 120).

The most substantial evidence to date of a dedicated dissimilatory iodate-reducing pathway has been found in *Pseudomonas* sp. SCT, *Aromatoleum toluclasticum* TC-10, and *Denitromonas iodatirespirans* (17, 24, 121). These studies demonstrate that the IO_3_^−^-reducing phenotype of *P. stutzeri*, *A. toluclasticum*, and *Denitromonas iodatirespirans* is due to a dedicated enzymatic pathway (17, 121, 122). The periplasmic fraction of the cell contained IO_3_^−^ reductase activity, and a follow-up study showed the presence of a unique DMSO reductase (IdrAB) related to the arsenite oxidase clade of DMSO reductases (17, 122). IdrAB appears to catalyze iodate reduction in strain SCT and *D. iodatirespirans* alongside two cytochrome C peroxidase-like proteins (IdrP_1_ and IdrP_2_), hypothesized to catalyze the removal of hydrogen peroxide generated via iodate reduction (17). Recently, Guo et al. demonstrated that HIO and H_2_O_2_ are intermediates of IO_3_^−^ reduction in *Shewanella* affirming the disproportionation model of IO_3_^−^ reduction in an analogous system (123, 124). While *Shewanella* does not have the idrAB-type iodate reductase, it is likely IO_3_^−^ reduction in organisms with an idrAB-type IO_3_^−^ reductases follows a similar chemistry. Furthermore, upon removing the *idrA* gene or molybdate from the media, IdrA activity ceases, demonstrating that *idrA* is required for IO_3_^−^ reduction and that the enzyme is molybdenum dependent (17). Together, advances in gene manipulation technologies and metagenomics have helped identify numerous mechanisms whereby microorganisms transform iodine oxyanions. Such observations may eventually shed further light on how these different iodate reduction mechanisms influence iodine geochemistry globally.

## CONSIDERATIONS FOR HOLISTICALLY DESCRIBING EMERGING ESOTERIC METABOLISMS

Suitable environmental cultures are pivotal to the characterization of DEMs (125). The fundamental principles of microbial cultivation and enrichment have been extensively reviewed previously (126), with modern strategies for cultivating fastidious organisms reviewed more recently (127). Several of these strategies were critical to the successful elucidation and characterization of DPO, DPR, and dissimilatory iodate reducer (DIR) diversity. Traditional selective enrichments sourced from environmental samples, a technique first established during the era of Winogradsky, served as the starting point for dissecting DPO, DPR, and DIR metabolisms (5). From carefully curated selective enrichments, an astute researcher can deploy a plethora of classical and advanced approaches to observe the presence of a particular metabolism and understand its underlying mechanisms. Below, we provide a set of guidelines and considerations for each of the steps in selective enrichment and analysis.

### Inoculum

The Baas Becking hypothesis (“everything is everywhere, but the environment selects”) suggests that any inoculum source eventually provides one with an active enrichment of the metabolism of interest. However, the choice of inoculum is important when one seeks to increase the likelihood of identifying an environmental representative microorganism performing a particular metabolism. In the provided examples, each system selected inoculum based on its likelihood to harbor the hypothesized metabolisms. For instance, wastewater digester sludge consistently yielded successful DPO enrichments compared to other sources. This sludge was chosen intentionally because of its high microbial loading, its underlying microbial diversity, and the frequent presence of phosphite in wastewater facilities (37, 128, 129). Likewise, the redox state of the inoculum plays a significant role in successful enrichments. For example, and perhaps counterintuitively because it is an anaerobic metabolism, DIR cultures used oxic marine sediments as enrichment sources since the formation of IO_3_^−^ is thermodynamically favored in more oxidizing environments (17). Integrating knowledge about known systems into potential inoculum sources ultimately allows for greater success during the enrichment process.

### Culture conditions

The environmental conditions that are needed to support a microorganism with a metabolism of interest are difficult to replicate in a laboratory setting, leading some to speculate that only a fraction of the world’s microorganisms have been cultivated (130). One way to overcome this challenge while establishing selective enrichments is to optimize the media design (131, 132). All organisms have four basic requirements: (i) an electron donor, (ii) an electron acceptor, (iii) an energy source, (iv) nutritional sources (carbon, nitrogen, sulfur, etc.). In some instances, the electron donor and energy source are independent of each other (e.g., phototrophs). Alternatively, an organism may produce its own electron acceptor (e.g., fermentative organisms reducing pyruvate) rather than utilizing an extracellular source. Among the multiple considerations, one must also account for temperature, pH, dissolved oxygen content, conductivity, redox state, pressure, salinity, and nutritional content (126). The nutritional content is the most challenging to parameterize, as environmental concentrations of certain nutrients are complex, transient, and may include undetectable quantities of essential substrates. Generalists without auxotrophies are among the easiest to enrich, as a defined media with an appropriate electron donor and acceptor adequately support growth. On the other hand, specialists may require media prepared by sterile-filtering extracted liquid from the host environment amended with supplemental nutrients, electron donors, and acceptors. Working with filtered environmental media is challenging because of the large quantities needed and the potential spatiotemporal variability of essential substrates at the source (133). When additional media are needed, experimental replication is complicated by changing environmental conditions that are hard to characterize and laborious to describe. Soil or sediment samples whose aqueous content is only a fraction of the sample volume would further exacerbate this challenge. Upon successfully defining the culture conditions, selective enrichments build upon standard enrichment practices with conditions favoring the growth of a specific organism (126). For the enrichment of the dissimilatory metabolisms described here, the electron donor and/or acceptor provide the selective pressure. Enrichments for DPOM amend phosphite as the only electron donor, energy source, and P source (25, 54, 55), whereas a non-fermentable electron donor (e.g., acetate or H_2_) and IO_3_^−^ or ClO_4_^−^ are used as acceptors to enrich for DIRM and DPRM, respectively (17, 134). Successful enrichments are validated by passaging the enrichment inoculum into fresh media, replicating previous observations, and ultimately using a suitable monitoring method to track growth and substrate turnover.

### Monitoring

Following the establishment of an enrichment culture, microbial growth must be measured to identify active enrichments consistently. Choosing which method to use for monitoring ultimately requires a compromise between throughput, accuracy, and precision. Historical methods, such as most probable number analysis (135, 136), direct cell counting (137), or dry cell weight measurements (138), were commonly used because of favorable economics, simplicity, and broad applicability. However, the advent of modern molecular techniques and high-throughput tools that overcome many of the drawbacks of these simple methods has relegated them to the ash heaps of history. For the aforementioned DEMs, optical density measurements were commonly used to measure turbidity using light at a wavelength equivalent to the size of a bacterial cell (600 nm). However, optical density is non-selective, can measure non-viable cells, does not properly account for the effect of cell morphology, and is further complicated in environmental enrichments by opaque sedimentary organics in the inoculum. Modern approaches such as quantitative PCR circumvent many of these challenges and can be used to monitor the growth proportional to the quantitative amplification of a marker gene from extracted DNA (139). Redundant primers for a phylogenetic marker gene (i.e., 16S ribosomal RNA gene) are typically used to measure community composition over time, but this is inadequate for identifying novel organisms whose taxonomy has not yet been defined. Primers can instead be designed to monitor the amplification of a physiological marker gene (i.e., *ptxD, idrA, pcrA*) whose abundance increases with its host. For metabolisms whose diversity has not yet been characterized, redundant primers capable of amplifying all unknown variants of a physiological marker gene would likely result in false negatives. Monitoring the consumption of an electron acceptor or donor that is directly involved in the enriched DEM can significantly enhance growth data. For instance, DPO, DIR, and DPR activities are measured using ion chromatography, as the metabolic redox reactions involved both consume and yield of inorganic ions that are compatible with this measurement technique (17, 54). Given that these DEMs proceed in a dissimilatory fashion, these data provide useful information on the activity of the enrichment culture, and whether it is actively metabolizing. While ion chromatography is sensitive and accurate when analyzing active enrichments, one often receives results after several hours or days. Colorimetric assays form an alternative that utilizes the reactivity of substrates of interest. For instance, iodate and iodide can be measured using the triiodide method, which relies on measuring the color change at 352 nm during the oxidation of iodide to triiodide under acidic conditions (140). Colorimetric approaches also have the advantage of being compatible with parallel analysis that can be performed with high-throughput automated strategies (141). Ultimately, monitoring an enrichment requires careful consideration of the chemistry potentiated by a particular pathway combined with orthologous data to support standalone observations. Combining several monitoring techniques enables one to describe potential energy flows and metabolic processes with increased detail.

### Isolation

The aim for most enrichments is to isolate an organism of interest and study it in an axenic culture. Pure cultures offer prime opportunities to analyze a metabolism in detail and study their mechanisms. Isolation of an organism is no facile task. Considering variables such as growth rate differences between community members, symbioses, nutrient requirements, and ability to grow on solid media is imperative to effectively establish axenic cultures. Methods such as dilution to extinction may prove useful when solid media isolation is not possible; however, it is prone to contamination with associated members of a microbial community or with different species within the same genus and is only effective when the target organism is the dominant member of the enriched community. Groups have optimized this method for the construction of simplified microbial communities (142). For scenarios where another organism consistently prevents the isolation of an axenic culture, knowledge of inhibitors or exclusionary secondary metabolites aids in isolation. For example, during the isolation of *Denitromonas iodatirespirans*, production of nitrite from denitrification aided in toxic inhibition of a contaminating *Vibrio* within the culture (17). While these methods describe the simplest approaches to isolation, other approaches are viable for those with sophisticated machinery. Fluorescence-activated cell sorting (with gene-specific antibody labeling) can aid in the isolation of community members harboring a certain gene (143). Similarly, optical tweezers or nutrient filter membranes can aid in picking individual cells that may have the phenotype of interest (144). Ultimately, the most significant challenge to isolating individual colonies is whether the organism of interest grows independently of any obligate symbioses. Cross-feeding in biofilms (145) and obligate syntropy between microorganisms (146) are other scenarios where even the most valiant attempt at developing defined media may fail. In considering the enrichment process, one might infer that its primary objective is isolation. However, failing to isolate an axenic culture does not sound the death knell for one’s endeavors, as comprehensive physiological analyses on microbial enrichments remain a potent tool for demystifying esoteric metabolic pathways.

### Genetics and genomics

Isolation attempts often provide a substantial amount of information about the dependencies of individual organisms hosting an esoteric metabolism. The methods used to describe the DEMs discussed in this perspective were characterized by two general approaches when understanding the genes potentiating a metabolism. Instances where isolation was fruitful enabled the use of more traditional molecular biology techniques (17). With the advent of whole-genome sequencing, one can expeditiously sequence organisms and utilize annotation software like Prokka or Prodigal to identify genes and provide genomic context to isolates (147, 148) Both DPR and DIR made use of these tools to identify target genes for each respective metabolism in the model organisms *Azospira suillum* sp. PS and *Denitromonas iodatirespirans* (17, 87). Sequencing the whole genome also provides a template for forward genetics screens such as RB-TnSeq (random barcode transposon sequencing: a method whereby genetic loci are perturbed through transposon mutagenesis and are subsequently mapped through sequencing) or random mutagenesis (149). A general advantage of the forward approach in investigating the underlying genes in these DEMs is that they identify a variety of axillary genes alongside the core genes (89). Additionally, approaches like RB-TnSeq may inadvertently identify community effects on a metabolism, leading to serendipitous observations such as the parasitic symbiosis between chlorate- and perchlorate-reducing microorganisms (84). Alternatively, with *a priori* knowledge of likely gene targets, one can attempt a targeted reverse genetics approach to knock out and evaluate genes associated with a particular metabolism. Groups researching DIR leveraged data suggesting that DMSO reductases likely support the metabolism. Phenotypic studies demonstrated a dependency on molybdate (17), a requirement for all DMSO reductases. Differential expression of proteins under iodate-reducing conditions and a comparative genomic analysis between DIRM and non-DIRM were used to identify the iodate reductase by reducing the list of possible gene targets (17, 122). Utilizing a reverse genetics approach ultimately demonstrated that targeted knockouts of the *idrA* gene lost DIR activity and confirmed that a dedicated DMSO reductase was necessary to enable DIR (17). Leveraging genomic data from isolates performing DEM of interest ultimately allows for researchers to begin understanding mechanisms at a much deeper level.

When enrichments fail to yield isolates, genome-resolved metagenomics is a powerful alternative for phylogenetically classifying a microbe of interest and predicting its metabolic requirements. The microbial biology of DPO presented a unique opportunity to deploy a metagenomic-centric strategy as only one pure culture of DPOM had been successfully acquired prior to the recent enrichment campaign (25, 54). Temporal sampling and metagenomic sequencing of enrichments allowed researchers to identify DPOM in complex communities based on gene content and binned taxa (where each bin represents the genome of a particular taxon) using differential abundance data (54). Using the dissimilatory *ptxD* as a marker gene, diverse DPOM were identified across these enrichment metagenomes (54). This newfound diversity expanded by 10-fold the known taxonomic hosts of DPO metabolism and captured the sequence diversity of DPO genes, which enabled the creation of profile hidden Markov models (pHMMs) (54, 150). Synergistically pairing metagenomics with more classical techniques often leads to a better understanding of the prevalence and diversity of novel metabolisms (130). Metagenomic analyses in DPOM relied on the analysis of proteomics data from a pure culture, for the attribution of biological activity to definitive gene sets to curate pHMMs (151). The recent isolation of *Phosphitispora fastidiosa,* only the second DPOM in axenic culture, validated this approach and the taxonomic diversity of DPO identified through metagenomic analysis (56). Similarly, studying DIRM genetics identified the necessity of the iodate reductase A subunit to the activity of DIR; these results provided the data required to create the pHMMs used to describe a novel ecological niche for DIRM at oxygen minimum zones globally (17). Hence, a combinatorial approach leveraging both classical wet lab techniques and newer computational approaches enables a targeted understanding of emerging esoteric metabolisms.

## CURATING AN INTEGRATIVE APPROACH TO DERIVE ENVIRONMENTAL RELEVANCE FROM BIOLOGICAL DATA

**Fig 1:**
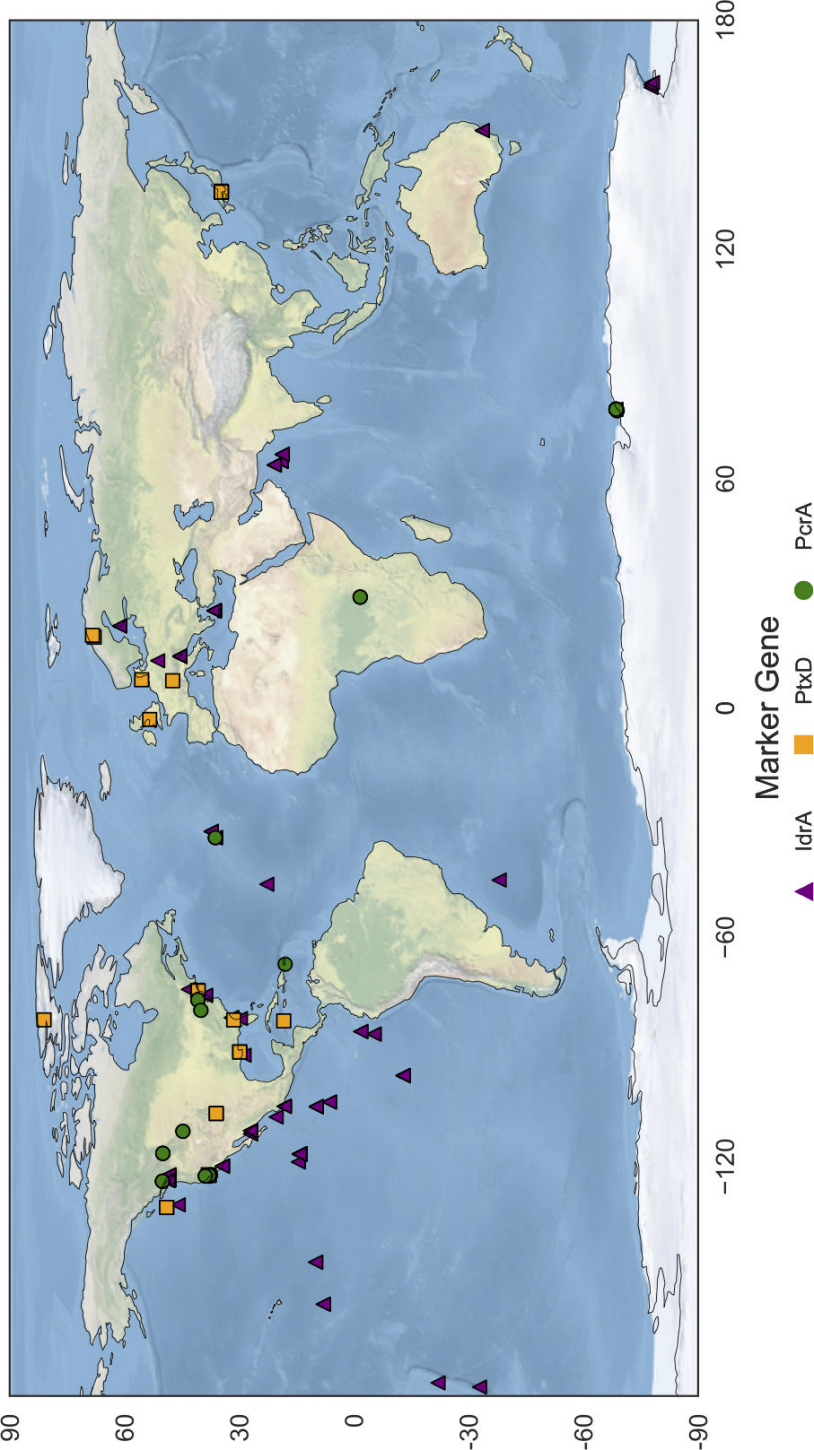
Distribution map of DIRM, DPRM, and DPOM. Using pHMMs of respective marker genes, sequence data from the JGI Integrated Microbial Genomes and Metagenomes database as presented by Nayfach et al*.* were mined for DIRM (IdrA), DPO (ptxD), and DPRM (PcrA) (152). Sequence metadata were used to plot the location on a global map in which genes were found, where each point is annotated with its corresponding marker gene (shape) and ecosystem type (color). Sequence data were produced by the U.S. Department of Energy Joint Genome Institute (http://www.jgi.doe.gov/).

**Fig 2:**
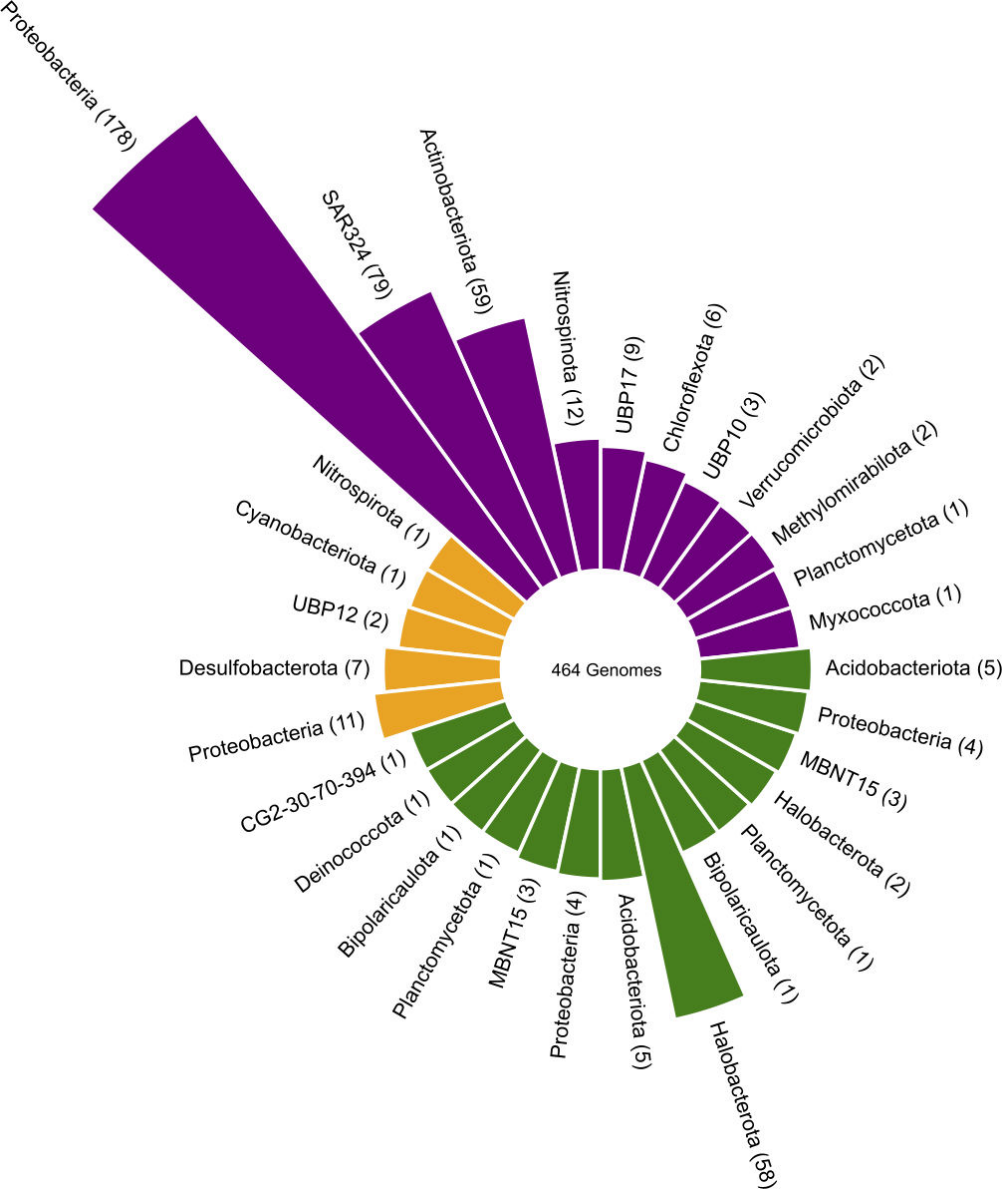
Phylogenetic distribution of marker genes associated with dark energy metabolisms. The phylogeny of DIRM, DPRM, and DPOM shown by absolute abundance of genomes belonging to each phylum. The pHMM for each respective marker gene was searched against the JGI Integrated Microbial Genomes and Metagenomes as compiled by Nayfach et al. (152). Metadata from any hits were used to extract phylum-level taxonomy. A total of 464 genomes contained one of these esoteric metabolisms.

The overarching goal of the studies of microbial perchlorate reduction, phosphite oxidation, and iodate reduction has been to apply an environmental microbial context to geochemical observations and paint a holistic portrait of the interface between biological and abiotic global systems. An integrated approach leveraging classical methods with computational tools parses public genome and metagenome databases for target metabolic genes, phylogenetic relationships, and gene neighborhood analyses (17, 54). One such analysis uses pHMMs to mine metagenomic data, capture the global distribution of the host metabolism, and provide insights into the ecological niche associated with a metabolism. By applying the pHMM search strategy for DPO, DPR, and DIR marker genes here, we found that DPO, DPR, and DIR (each of which are still considered esoteric metabolisms) are in fact globally prevalent and distributed broadly (Fig. 1 and 2).

Biological signals that identify the location of particular metabolisms carry implications for the redox activity of the iodine, chlorine, and phosphorous elemental cycles based on distribution patterns of each metabolism. For instance, there exists a paucity of mechanisms describing the biological contribution to the IO_3_^−^/I^−^ disequilibrium globally. However, by identifying biological signals indicative of DIR, we add necessary context to the geochemical observations describing the IO_3_^−^/I^−^ disequilibrium (17, 112, 153). Similarly, sampling diverse environments with a holistic understanding of the environment’s geochemistry helps identify other locations where these DEMs hide. A recent study looking at a methane-oxidizing biofilm under iodine-rich formation water identified a metagenome-assembled genome of a putative methane oxidizing iodate-reducing microorganism (141). Drawing further connections between DIR and cofactor requirements, enzymatic activity, and microbial lifestyle enables modelers and geochemists to precisely parameterize the geochemical observations. Thus, through additional parameterization, one can analyze the likelihood of iodine cycling at a particular location and more accurately predict the downstream effects of this understudied metabolism.

The biological signature of DPRM distribution paints a picture of where chlorine redox cycling likely occurs. Our data corroborate observations that DPRM are ubiquitous and are found in diverse environments ranging from contaminated wastewater to pristine soils and sediments (78). The broad distribution of DPRM shows the genetic potential for (per)chlorate removal globally and provides biological indicators of locations where (per)chlorate concentrations may be higher, such as thermal springs or certain freshwater environments (Fig. 1). Metagenomic surveys identified the genomic potential for chlorine cycling in Arctic coastal tundra soils, with genes for perchlorate reduction, haloperoxidases, halogenases, and reductive dehalogenases found at multiple basins (154). Identifying genes associated with DPR at these locations indicates that selective pressures have enabled the maintenance of these genes and possibly point to a source of (per)chlorate cycling.

Combining metagenomic signals for DPOM with existing geochemical signals for reduced phosphorous could cooperatively identify hot spots for microbial phosphorous redox cycling. For example, wastewater digester sludge and wetland sediments are both environments where phosphite and DPOM have been independently identified, jointly providing a signal for environmental phosphorous redox activity (Table 2). The prevalence of *Desulfomonilia_A* DPOM in wastewater digestate is a pronounced observation across continents (54), implying a role for phosphorous redox activity within the anthropogenic waste management stream. Prevailing waste management practices have been criticized for unsustainably flushing limited phosphorous resources into the ocean (155), and this challenge could be accentuated by highly soluble reduced phosphorous compounds such as phosphite (1,000× more soluble than phosphate) (156). Meanwhile, novel sites of DPOM activity are likely to be found in environments with detectable levels of phosphite where DPOM have not yet been identified (e.g., rivers, lakes, and geothermal water) (Table 2). Because the detection of phosphine frequently indicates the presence of other reduced phosphorous compounds (e.g., phosphite), any reduced phosphorous compound might serve as a bellwether for the potential presence of DPOM. Subsurface groundwater is the only environment to date in which DPOM have been detected but reduced phosphorous has not, and this environment may exemplify cryptic redox cycling of phosphorous (Table 2). As is descriptive of reduced phosphorous compounds, the substrates of cryptic cycles remain undetectably low or static due to microbial turnover and frequently represent geochemical intermediates with poorly understood redox chemistry (157). The subsurface was highlighted by Figueroa et al. to be a likely location for phosphite neogenesis and a promising ecosystem for DPOM (31), and Ruttenberg et al. have emphasized the subsurface as an important black box for the transport of phosphorous to marine and oceanic systems (29). DPOM in the subsurface may consequently be critical in subsurface phosphorous cycling while also performing primary production in this aphotic and energy-limited environment (31).

**TABLE 2:** Literature review of the distribution of DPOM and reduced phosphorus species^*c*^

Environment	DPOM	Phosphite	Hypophosphite	Phosphine	Phosphonates
Deep subsurface aquifer**/**groundwater	(117)	n.a.^*a*^	n.a.	n.a.^*a*^	n.a.
Engineered systems	(25, 117)	(158)	n.a.	(42)	(159)
Biological wastewater digestor sludge	(117)	(160)	n.a.	(42, 161)	(162)
Industrial wastewater (petrochemical**/**landfill)	(117)	(36)	(36)	(42, 163)	(164)
Freshwater sediments**/**wetlands**/**marsh	(117)	(165)	(165)	(42, 161, 166, 167)	(168)
Marine wetlands**/**saltmarsh**/**sediment	Fig. 1	n.a.	n.a.	(42, 51, 166)	(169)
Terrestrial soils	(117) and Fig. 1	(41, 170)	(41, 170)	(163, 171)	(172)
Extraterrestrial	n.a.	(173)	(173)	(42, 43, 173)	(174)
Geothermal**/**hydrothermal water	n.a.	(175)	n.a.^*b*^	n.a.	n.a.
Lake water	n.a.	(160)	n.a.	(42)	(176)
River water	n.a.	(165)	(165)	(42)	(162)
Sea water	n.a.	n.a.	n.a.	(166)	(177–179)
Animal gut**/**excrement	n.a.	(180)	(180)	(42)	n.a.

^*a*^ Reduced phosphorus species have been detected from the groundwater of river basins but not in deep subsurface systems (165).

^*b*^ Although hypophosphite was not measured in geothermal or hydrothermal systems, Pech et al. hypothesize that it is likely to be present.

^*c*^ For each environment listed in the first column, references for the detection of reduced phosphorus species or DPOM are listed numerically in the subsequent columns. The black box highlights those environments in which reduced phosphorus has been identified but DPOM have not.

## CONCLUSIONS

Designing geochemical studies and microbial physiology studies with an interdisciplinary perspective opens the door for a more rigorous approach that optimizes the applicability of both fields to each other. Providing genetic and molecular data around individual metabolisms avoids overfitting data by validating gene function and activity. Likewise, using computational predictions to understand where certain metabolisms persist enables the parameterization of global elemental cycles and provides greater precision in describing global elemental transport. Combining data from both fields into individual studies allows researchers to consciously reject unfeasible hypotheses and propose mechanisms that are applicable environmentally and in the lab. Our discussion reviews studies that utilize a harmonized framework to understand esoteric metabolisms and proposes some “best practices” for integrating microbial physiology to elemental transport phenomena. We describe how such a framework enables the interpretation of environmental data and propose that further metagenomic surveys include geochemical data and molecular biology tools for validation. Such an interdisciplinary approach enables a rigorous method by which we may understand the interface of microbial systems within our world.
